# Receptive Field Sizes of *Nyx^nob^* Mouse Retinal Ganglion Cells

**DOI:** 10.3390/ijms23063202

**Published:** 2022-03-16

**Authors:** Maj-Britt Hölzel, Marcus H. C. Howlett, Maarten Kamermans

**Affiliations:** 1 Netherlands Institute for Neuroscience Amsterdam, 1105 BA Amsterdam, The Netherlands; m.holzel@nin.knaw.nl (M.-B.H.); m.howlett@nin.knaw.nl (M.H.C.H.); 2 Department of Biomedical Physics, Academic Medical Center, University of Amsterdam, 1105 AZ Amsterdam, The Netherlands

**Keywords:** receptive field, retina, retinal ganglion cells (RGCs), *Nyx^nob^*, congenital nystagmus, CSNB, green-light preferring RGCs, UV-light preferring RGCs

## Abstract

Patients with congenital nystagmus, involuntary eye movements, often have a reduced visual acuity. Some of these patients have a retinal-specific mutation in the protein nyctalopin, which is also present in the *Nyx^nob^* mouse. In these mice, retinal ganglion cells (RGCs) have oscillatory activity, which leads to expanded axonal projections towards the dLGN and consequently to a desegregation of retinal projections to the brain. In this study, we investigate whether the receptive fields of *Nyx^nob^* RGCs have also expanded by measuring the size of their receptive fields using MEA recordings. Contrary to our expectation, relative to wild-type (WT) mice we found receptive field sizes in the *Nyx^nob^* retina had not increased but instead had decreased for green-light preferring RGCs. Additionally, we also found the receptive fields of UV-light preferring RGCs are larger than green-light preferring RGCs in both WT and *Nyx^nob^* mice.

## 1. Introduction

Shortly after birth, patients with congenital nystagmus develop involuntary oscillating eye movements, i.e.,: nystagmus [1,2,3]. Additionally, most of these patients have a low visual acuity that can be as low as 1.0 logMar [3,4]. Surgical interventions temporally stop the nystagmus, but the visual acuity hardly improves [5,6,7]. This suggests that the nystagmus may not be solely responsible for the low visual acuity in these patients.

Recently we showed that *Nyx^nob^* mice, a model for congenital stationary night blindness (CSNB; [8,9]), have pendular nystagmus, just as patients with mutations in the human orthologue gene *NYX* [10,11,12]. In these mice, the activity of many retinal ganglion cells (RGCs) synchronously oscillates. This oscillatory activity is sent to the accessory optic system (AOS) where it induces oscillatory eye movements i.e., nystagmus. A previous study reported that in *Nyx^nob^* mice, RGC projections to the dorsal lateral geniculate nucleus (dLGN) desegregate after eye opening [13]. Normally the RGC projections of the two eyes target separate areas in the dLGN, but in *Nyx^nob^* mice these projection areas overlap. This raises the possibility that the synchronized oscillatory activity of RGCs may disturb the normal development of their projections to the dLGN, thereby reducing the visual acuity.

Since the projection area of *Nyx^nob^* RGCs in the dLGN is larger compared to WT mice [13], we asked if similar changes already occur in the retina. Therefore, we performed multi-electrode array recordings of RGCs from whole-mount retinas of WT and *Nyx^nob^* mice, and measured their receptive field size using a random checkerboard stimulus. We found the receptive fields of *Nyx^nob^* RGCs were not increased but were instead slightly smaller in green-light preferring RGCs. Furthermore, we also found the receptive field sizes of UV-light preferring RGCs were larger than those of green-light preferring RGCs. These results suggest that retinal changes in receptive field size of RGCs do not underly the low visual acuity associated with nystagmus in CSNB.

## 2. Results

### 2.1. Nyx^nob^ RGCs Are Spontaneously Oscillating

**Figure 1:**
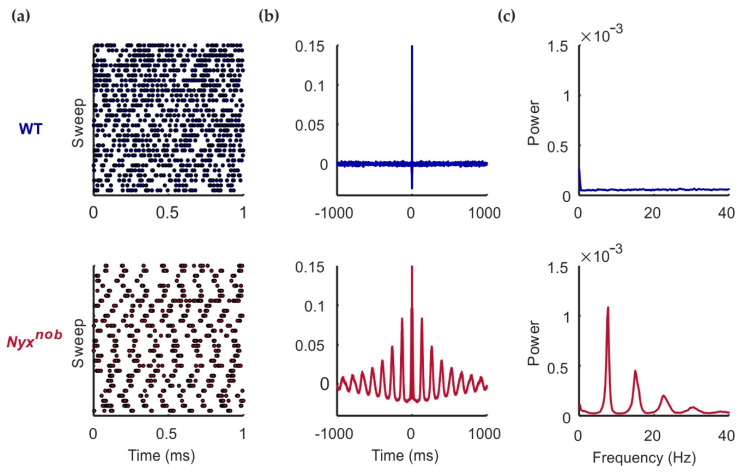
RGC oscillations in the *Nyx^nob^* retina. Spontaneous spiking activity in the dark of an example WT (blue), and *Nyx^nob^* (red) RGC. (**a**) Raster plots show the spontaneous spiking activity, in which each dot represents a spike. The example *Nyx^nob^* RGC shows clear oscillatory firing behavior. (**b**) The autocorrelation of the *Nyx^nob^* RGC shows periodic variations, which is an indication of oscillatory firing. (**c**) The power spectrum of this representative example *Nyx^nob^* RGC shows a peak with the fundamental frequency of 7.8 Hz and harmonics. The WT RGC power spectrum has no such peaks.

Previously we reported that retinal ganglion cells (RGCs) of *Nyx^nob^* mice fire spontaneous action potentials in an oscillatory manner [10]. We wanted to confirm if this was also the case in this current study. Using a multi-electrode array, we made extracellular recordings of the spontaneous spiking activity of WT and *Nyx^nob^* RGCs (Figure 1a). The spontaneous activity of approximately 48% of *Nyx^nob^* RGCs showed oscillatory firing behavior, as indicated by the presence of periodic variations in their autocorrelation (Figure 1b, bottom panel) as well as by prominent peaks in their power spectrum (Figure 1c, bottom panel). On average, the activity of these RGCs oscillated at a frequency of 7.7 ± 0.08 Hz (mean ± SEM; n = 301 RGCs, N = 7 mice). For two of the seven *Nyx^nob^* retinas, very few RGCs oscillated (4 of 162 RGCs). Excluding these two retinas, 64% of RGCs from the remaining five retinas oscillated (mean oscillation frequency: 7.6 ± 0.07 Hz; n = 297 RGCs, N = 5 mice). For the WT retina, we found no indications of oscillating spontaneous RGC activity (Figure 1, top panel).

### 2.2. Nyx^nob^ Retinas Lack Light-Evoked ON-Responses

**Figure 2:**
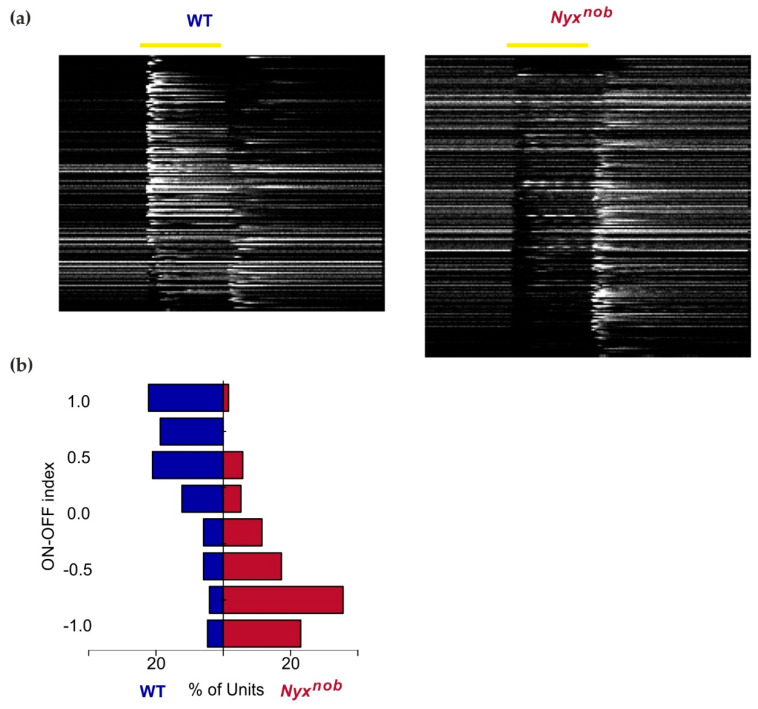
Absent ON-responses in the *Nyx^nob^* retina. (**a**) Heatmap of the average responses of WT (left, n = 171 RGCs) and *Nyx^nob^* (right, n = 191 RGCs) RGCs to a 500 ms light flash. Each row represents the average response of a single RGC, ranked in descending ON-OFF index order. WT retinas contain mainly ON-RGCs, which are mostly absent in the *Nyx^nob^* mouse. Instead, *Nyx^nob^* RGCs show increased firing at the light flash offset. In addition, many *Nyx^nob^* RGCs show late-onset oscillatory-firing behavior during the light flash. (**b**) The ON-OFF indices of RGCs are shown in (**a**). The comparison shows a clear shift towards OFF-responses in the *Nyx^nob^* mouse (ON-OFF index of 1 indicates a pure ON-response and −1 for a pure OFF response).

Since the *Nyx^nob^* mutation renders the ON-pathway non-functional, light-evoked ON responses are absent in the *Nyx^nob^* retina [8,10,14]. To confirm that this was also the case here, we measured RGC responses to a 500 ms light flash. Figure 2a shows the activity of the RGCs in the WT and *Nyx^nob^* retinas. Each line represents the mean response to a 500 ms white-light flash of one RGC. The WT retinas contain mainly ON-RGCs as indicated by their increase in firing at the onset of the light flash (Figure 2a-left, n = 171). In contrast, ON responses are largely absent in *Nyx^nob^* mice (Figure 2a-right, n = 191). Instead, most RGCs in the *Nyx^nob^* retina are OFF-RGCs. They stop firing at the stimulus onset and increase their firing rate after the light flash offset. Furthermore, many *Nyx^nob^* RGCs show oscillatory firing behavior commencing soon after the onset of the light stimulus. That these oscillations are apparent in the RGC’s mean response indicates that they are being phase-reset by the onset of the light stimulus, which is consistent with our previous reports [10]. To quantify the change of light responsiveness in the *Nyx^nob^* retina compared to WT, we calculated the ON-OFF index for the light responses. Figure 2b shows that there is a clear shift towards OFF-responses (negative ON-OFF-index) in the *Nyx^nob^* compared to the WT retina.

### 2.3. RGC Receptive Field Sizes Are Similar in WT and Nyx^nob^

**Figure 3:**
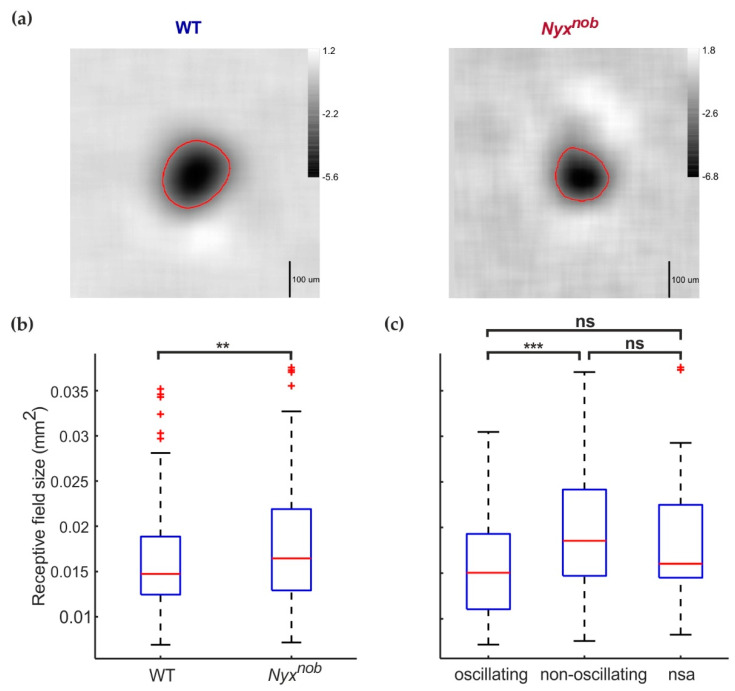
Receptive field size increased in the *Nyx^nob^* retina. (**a**) The reverse spike-triggered average receptive-field estimates for WT (left) and *Nyx^nob^* (right) green-preferring RGCs. The red circles indicate the 40% contour line, whose 2D area was used as our measure of receptive field size. (**b**) Boxplots depicting the distribution of receptive field sizes for WT and *Nyx^nob^* retinas. RGCs from *Nyx^nob^* retinas (n = 189 from 7 mice) generally had larger receptive field sizes compared to WT RGCs (n = 172 from 5 mice) (**c**) Boxplots of receptive field sizes of oscillating and non-oscillating *Nyx^nob^* RGCs. The receptive field size of non-oscillating RGCs (n = 95 RGCs from 7 mice) is increased compared to oscillating RGCs (n = 63 RGCs from 7 mice). The median receptive field size of *Nyx^nob^* RGCs displaying no spontaneous activity (nsa, n = 31 from 7 mice) was located between that of oscillating and non-oscillating RGCs. ns: not significant, ** *p* < 0.01, *** *p* < 0.001.

Next, we determined the receptive field size of RGCs in the WT and *Nyx^nob^* retina. We mapped the receptive field of RGCs by generating reverse spike-triggered averages using a temporal-spatial modulated white-noise checkerboard stimulus consisting of non-overlapping green and UV checkers (Figure 3a). On average, we mapped less receptive fields per retina for the *Nyx^nob^* (21.6 receptive fields per retina) compared to the WT (34.4 receptive fields per retina). We next determined the receptive field size, measured here as the surface area of the contour corresponding to 40% of a receptive field’s greatest baseline-to-peak amplitude (Figure 3a, red lines). Figure 3b shows the distribution of receptive field sizes for WT and *Nyx^nob^* RGCs, depicted as boxplots. On average, the receptive field size was larger for *Nyx^nob^* mice than for WT mice (Figure 3b; median ± MAD; WT: 0.015 ± 0.0032 mm^2^, n = 172 RGCs; *Nyx^nob^*: 0.016 ± 0.0043 mm^2^, n = 189 RGCs; Mann–Whitney U, *p* = 0.009).

In the *Nyx^nob^*, the spontaneous spiking activity of some, but not all, RGCs oscillate. Do the oscillating and non-oscillating *Nyx^nob^* RGCs differ in terms of their receptive field size? To test this, we compared the receptive field size of oscillating and non-oscillating RGCs (Figure 3c). Of the 189 *Nyx^nob^* RGCs for which the receptive field size could be reliably measured, 63 RGCs oscillated with a mean (±SEM) oscillation frequency of 7.7 ± 0.15 Hz and 95 RGCs did not oscillate. For the remaining 31 RGCs, we could not reliably determine if they were oscillatory or not as they did not spike during the period when we recorded spontaneous activity. Surprisingly, receptive field sizes of non-oscillating RGCs where larger than for the oscillating RGCs (Figure 3c; median ± MAD; oscillating: 0.015 ± 0.0041 mm^2^, n = 63 RGCs; non-oscillating: 0.019 ± 0.0046 mm^2^, n = 95 RGCs; Mann–Whitney U, *p* = 0.000). The median (±MAD) receptive field size of the 31 RGCs with no spontaneous activity lays between that of the oscillating and non-oscillating RGCs (Figure 3c, 0.016 ± 0.0033 mm^2^, n = 31), possibly indicating this group included both oscillating and non-oscillating RGCs.

**Figure 4:**
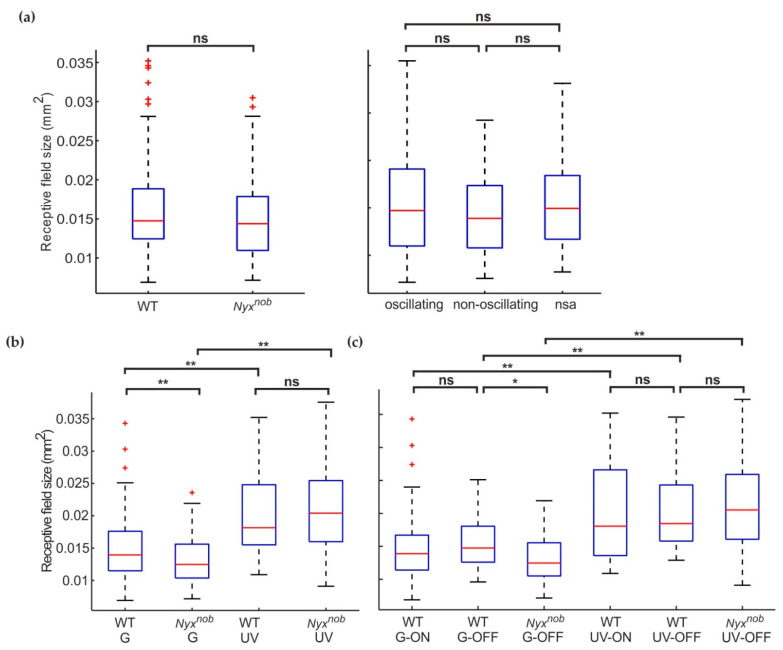
A closer examination reveals green preferring RGC receptive fields are smaller in the *Nyx^nob^* retina. (**a**) Two of the seven *Nyx^nob^* retinas showed almost no oscillatory spiking behavior. Excluding RGC data from these two retinas changed two outcomes presented in Figure 3b,c. Firstly (left), as demonstrated by their boxplots, the receptive field sizes of WT (n = 172 from 5 mice) and the remaining *Nyx^nob^* (n = 108 from 5 mice) RGCs no longer differed. Secondly (right), the remaining *Nyx^nob^* oscillating (n = 62 from 5 mice) and *Nyx^nob^* non-oscillating (n = 31 from 5 mice) RGCs did not differ in terms of receptive fields sizes, as shown by their boxplots. (**b**) Splitting the receptive field sizes according to their spectral sensitivity revealed green-preferring *Nyx^nob^* RGCs (*Nyx^nob^*-G: n = 75 from 7 mice) had smaller receptive field sizes than did green-preferring WT RGCs (WT-G: n = 134 from 5 mice). For UV-preferring RGCs, there was no difference in receptive field size between WT (WT-UV: n = 38 from 5 mice) and *Nyx^nob^* (*Nyx^nob^*-UV: n = 114 from 7 mice). Additionally, green-preferring RGCs have a smaller receptive field than UV-sensitive RGCs in both WT and *Nyx^nob^* retinas (see results for details). (**c**) Boxplots comparing the receptive field size of WT ON- and OFF- RGCs, as well as Nyxnob OFF RGCs, separated based on their spectral sensitivity (WT-G-ON: n = 90; WT-G-OFF n = 44; WT-UV-ON: n = 18, WT-UV-OFF: n = 20 from 5 mice; *Nyx^nob^*-G-OFF: n = 61: *Nyx^nob^*-UV-OFF: n = 97). There was no difference between WT ON- and OFF- RGCs but consistent with (**b**) UV-preferring RGCs have a larger receptive field than green-preferring RGCs for both, WT and *Nyx^nob^* RGCs. nsa: no spontaneous activity ns: not significant, * *p* < 0.05, ** *p* < 0.01.

As previously mentioned, for two *Nyx^nob^* retinas less than 3% of measured RGCs displayed oscillating spontaneous activity, which is significantly less than in the other retinas. To test if including data from these two *Nyx^nob^* retinas was affecting the receptive field sizes (comparisons shown in Figure 3b,c), we excluded them and repeated the analyses (Figure 4a). When we did so, we found no difference in receptive field sizes between *Nyx^nob^* and WT RGCs (Figure 4a-left, medians ± MAD, WT: 0.015 ± 0.0032 mm^2^, n = 172 RGCs; *Nyx^nob^*: 0.014 ± 0.0034 mm^2^, n = 108 RGCs; Mann–Whitney U, *p* = 0.105), nor between oscillating and non-oscillation RGCs in the *Nyx^nob^* retina (Figure 4a-right, medians ± MAD, oscillating: 0.015 ± 0.0037 mm^2^, n = 62 RGCs; non-oscillating: 0.014 ± 0.0033 mm^2^, n = 31 RGCs; Mann–Whitney U, *p* = 0.364).

We next asked why the data from these two atypical *Nyx^nob^* retinas has such a pronounced effect on the comparison of receptive field sizes. To investigate this further, we grouped the receptive field sizes by their corresponding RGC’s spectral preference Figure 4b). From the two *Nyx^nob^* retinas with very few oscillating RGCs, 81 RGCs generated reliable reverse spike-triggered averages. Of these, the majority were classed as UV-light preferring RGCs (UV: 96% (78 of 81 RGCs), green: 4% (3 of 81 RGCs)). For the remaining five *Nyx^nob^* retinas, we found two times more green- than UV-light preferring RGCs (green: 67% (72 of 108 RGCs); UV: 33% (36 of 108 RGCs)), which was similar to the WT retinas (green: 78% (134 of 172 RGCs); UV: 22% (38 of 172 RGCs)).

### 2.4. Green-Light Preferring RGCs Have Smaller Receptive Fields than Do UV-Light Preferring RGCs

On average, the receptive field size of green-light preferring RGCs (Figure 4b) was smaller than for UV-light preferring RGCs in WT (median ± MAD; WT-G: 0.014 ± 0.0027 mm^2^, n = 134; WT-UV: 0.018 ± 0.0044 mm^2^, n = 38; Mann–Whitney U, *p* = 0.000) and in the *Nyx^nob^* mouse (median ± MAD; *Nyx^nob^*-G: 0.013 ± 0.0025 mm^2^, n = 75; *Nyx^nob^*-UV: 0.020 ± 0.0048 mm^2^, n = 114; Mann–Whitney-U, *p* = 0.000). The abundance of UV cells in the two atypical *Nyx^nob^* retinas may have biased our receptive field size estimates, making it appear as if they were generally larger in *Nyx^nob^* retinas.

### 2.5. Smaller Receptive Fields of Green-Light Preferring Nyx^nob^ RGCs Compared to WT

For UV-light preferring RGCs, there was no difference in receptive field size between WT and *Nyx^nob^* (Figure 4b-right, median ± MAD; WT-UV: 0.018 ± 0.0044 mm^2^, n = 38; *Nyx^nob^* -UV: 0.020 ± 0.0048 mm^2^, n = 114; Mann–Whitney U, *p* = 0.267). Surprisingly however, green-light preferring RGCs did have a somewhat smaller receptive field size in the *Nyx^nob^* compared to WT (Figure 4b-left, median ± MAD; WT-G: 0.014 ± 0.0027 mm^2^, n = 134; *Nyx^nob^* -G: 0.013 ± 0.0025 mm^2^, n = 75; Mann–Whitney U, *p* = 0.003).

### 2.6. WT ON- and OFF-RGCs Have Similar Receptive Field Sizes

A possible reason for this difference in green-lighting preferring RGCs could be that the ON receptive fields are larger than the OFF receptive fields. As *Nyx^nob^* mice have lost their ON-RGCs this may have resulted in only smaller OFF-RGC receptive field sizes being measured whereas WT mice measurements would consist of a mixture of larger ON-, and smaller OFF-, RGC receptive fields. To test this, we next compared the receptive field sizes of WT ON- and OFF-RGCs separately for green- and UV-light preferring RGCs (Figure 4c). We found no differences between green- ON and green-OFF-RGCs (WT-G-ON: 0.014 ± 0.0025 mm^2^, n = 90 RGCs; WT-G-OFF: 0.015 ± 0.0030 mm^2^, n = 44 RGCs; Mann–Whitney U, *p* = 0.313) or between UV-ON and UV-OFFs (WT-UV-ON: 0.018 ± 0.0046 mm^2^, n = 18 RGCs; WT-UV-OFF: 0.019 ± 0.0037 mm^2^, n = 20 RGCs; Mann–Whitney U, *p* = 0.675). Therefore, the missing ON- RGCs in the *Nyx^nob^* retina do not account for their green-light preferring RGC’s smaller receptive field size. In addition, UV- ON and OFF receptive field sizes are respectively larger than those of G- ON and OFF RGCs (ON: Mann–Whitney U, *p* = 0.003, OFF: Mann–Whitney U, *p* = 0.002), which is consistent with our findings shown in Figure 4b. This is also the case for *Nyx^nob^* G-OFF and UV-OFF RGCs (*Nyx^nob^* UV-ON: 0.013 ± 0.0025 mm^2^, n = 61 RGCs; *Nyx^nob^* -UV-OFF: 0.021 ± 0.0049 mm^2^, n = 97 RGCs; Mann–Whitney U, *p* = 0.000). Furthermore, receptive fields of *Nyx^nob^* G-OFF RGCs are smaller than those of WT (Mann–Whitney U, *p* = 0.005) while being of similar size for *Nyx^nob^* and WT UV-OFF RGCs (Mann–Whitney U, *p* = 0.417).

## 3. Discussion

Contrary to our expectations, we found that RGC receptive field sizes in *Nyx^nob^* mice are not larger compared to WT but are in fact smaller, at least for green-light preferring RGCs. In contrast, we discerned no receptive field size difference between WT and *Nyx^nob^* UV-light preferring RGCs. Furthermore, we found UV-light preferring RGCs in both WT and *Nyx^nob^* had larger receptive field sizes than did green-light preferring RGCs, and we did not find a difference in the receptive field sizes of ON- and OFF-RGCs in the WT.

In this study, and despite the oscillatory firing of many RGCs, we were able to successfully measure the receptive fields of *Nyx^nob^* RGCs using a random checkerboard stimulus. However, there were on average fewer receptive fields determined per retina in the *Nyx^nob^* mouse compared to WT. This lower success rate may reflect the absence of ON-RGCs receptive fields in the *Nyx^nob^* mouse. The oscillatory firing may have also contributed, by inducing additional noise thereby reducing the likelihood a receptive field measurement passed our inclusion criteria.

We found green-sensitive RGC receptive field sizes were decreased in the *Nyx^nob^* retina compared to WT. Since *Nyx^nob^* mice lack ON-RGCs, this difference may have occurred if ON-RGCs have larger receptive fields than OFF-RGCs. However, when we tested for this possibility, we found no difference in receptive field size between ON- and OFF-RGCs, for either green- or UV-light preferring RGCs. This is consistent with previous reports [15,16] that also showed ON- and OFF- RGCs have the same receptive field sizes in the adult mouse retina. On the other hand, another study showed OFF-RGCs have larger receptive fields than ON-RGCs [17]. Since we and others find ON receptive fields are equal to or smaller than OFF receptive fields, a lack of ON-RGCs cannot account for the decrease in receptive field size we find for *Nyx^nob^* green-sensitive RGCs.

The receptive field sizes of the green-sensitive RGCs may have been changed by RGC oscillation-induced plasticity. In *Nyx^nob^* mice, the axons of RGCs project to the dLGN over a wider area, presumably due to their spontaneous oscillatory activity [13]. Although we did not measure RGC dendritic field morphology, we assume the sizes of the dendritic and receptive fields are related to each other and as such receptive field size is a proxy measure of dendritic field size. This implies that, unlike the axonal projections, the dendritic integration area of the green-sensitive RGC did not increase but instead decreased. If true it suggests RGC oscillation-induced plasticity has differential effects on the dendritic and axonal trees: The spatial extent of the dendritic arbor decreases while it increases for the axonal tree.

A change in the balance between the excitatory-center and inhibitory-surround may have also led to green-light preferring *Nyx^nob^* RGCs having smaller receptive field sizes. Either an increase in surround inhibition or a decrease in center excitation would result in reduced receptive field size. While we cannot rule out either, increased surround inhibition is the more parsimonious explanation.

One intriguing aspect of our study was the observation that UV-light preferring RGCs on average had larger receptive field sizes than did green-light preferring RGCs. To the best of our knowledge, this is the first report of such a difference in mice. This asymmetry may reflect different ecological demands placed on the two spectral systems. The mouse retina is divided into two spectral areas- a dorsal green region monitoring the lower visual field (ground) and a ventral UV part directed towards the upper visual field of the mouse (sky) [18,19]. As the sky has less spatial detail, RGCs presumably do not require small receptive fields. However, the visual scene on the ground does have a higher spatial frequency content and so smaller receptive fields may be more efficient for resolving fine details in the mouse’s surroundings.

## 4. Materials and Methods

### 4.1. Animals

All experimental animals were in a *SPIG1^+^* background [20]. For this study, five male SPIG1^+^ (referred to here as WT) and seven *Nyx^nob^*/*SPIG1^+^* mice were used with an age ranging between 5 and 21 weeks. The SPIG1^+^ mice were obtained from the Noda lab (National Institute for Basic Biology, Okazaki, Japan) and the *Nxy^nob^* mice from the McCall lab (University of Louisville, Louisville, KY, USA). All animal experiments were carried out under the responsibility of the ethical committee of the Royal Netherlands Academy of Arts and Sciences (KNAW) acting in accordance with the European Communities Council Directive of 22 July 2003, (2003/65/CE). The experiments were performed under the license number AVD-801002016517, issued by the Central Comity Animal Experiments of the Netherlands.

### 4.2. Retinal Dissection

Retinal isolation for MEA recordings was performed as described earlier in [10]. Briefly, mice were first dark-adapted for at least an hour, sedated using a mixture of CO_2_/O_2_, and then euthanized by cervical dislocation. Under dim red light, eyes were removed, then in room temperature Ames’ Medium (Sigma-Aldrich, St Louis, MO, USA) the anterior segment and vitreous humor removed and the retina isolated from the remaining eyecup. Four shallow equidistant radial cuts were made to the isolated retina, after which it was flat-mounted on a filter paper annulus (1 mm inner radius; 0.8 µm hydrophilic MCE MF-MilliporeTM membrane filter, Merck Millipore Ltd., Tullagreen, Ireland) then placed photoreceptor side up on a perforated 60-electrode MEA chip (60pMEA200/30iR-Ti, using a MEA2100 system: Multichannel Systems, Reutlingen, Germany) in a recording chamber mounted on a Nikon Optiphot-2 upright microscope and viewed under IR with an Olympus 2x objective and video camera (Abus TVCC 20530). The recording chamber was continuously superfused with Ames’ medium gassed with a mixture of O_2_ and CO_2_ at a pH of 7.4 and a temperature of 29–36 °C. All retinas were acclimatized to these conditions, in the dark, for at least 15 min before recordings commenced.

### 4.3. Data Acquisition

The extracellular RGC activity of WT and *Nyx^nob^* mice was recorded as described previously in [10]. MEA data was acquired using MC rack (Multichannel Systems, Reutlingen, Germany) at a sampling frequency of 25 kHz, zero-phase bandpass filtered (250–6250 Hz) with a fourth-order Butterworth filter in Matlab (Mathworks, Natick, MA, USA), then sorted into single-unit activity with Plexon offline sorter (Plexon, Dallas, TX, USA). Spikes were detected using an amplitude threshold > 4*σ_n_* where *σ_n_* is an estimation of the background noise with *x* being the bandpass-filtered signal [21].

(1) $$\sigma_{n}=median\left(\frac{\left|x\right|}{0.6745}\right)$$

The detected spikes were manually sorted into single units based on the first two principal components versus time.

### 4.4. Optical Stimulator

Light stimuli were generated using Psychophysics Toolbox Version 3 [22] and projected onto the retina from the photoreceptor side by a custom-modified DLP projector (Light Crafter 4500, Wintech, Carlsbad, CA, USA) using a custom-built 2x water immersion objective. The light source of the DLP was replaced by a 4 channel LED light source from Thorlabs (4 colors LED Head, Thorlabs, Germany). The four LEDs had peak wavelengths of 625 (red), 530 (green), 455 (blue), and 385 nm (UV) and were driven by a custom-made LED driver. While color is a higher-order and species-dependent percept, for simplicity we refer to the light emitted by the four LEDs respectively as red, green, blue, and UV. An optical feedback loop ensured linearity between the driver current and the light output of the LEDs. The Light Crafter was used in “Pattern mode” such that each frame consisted of 4 subframes (R, G, B, UV) each coded in 6 bit per pixel. The presentation time of each subframe was 4.1 ms resulting in a frame rate of about 60 Hz. For the present study, the red and the blue LEDs were turned off. The maximal output on the retinal surface of the green- and UV- LEDs were respectively 1.76 × 1018 quanta m^−2^s^−1^ and 1.76 × 1018 quanta m^−2^s^−1^. White light stimuli consisted of the equal quantal output of the green- and UV-LEDs.

Full-field light flashes and a white-noise spatial-temporal modulated checkerboard were used as light stimuli. The full field stimulus consisted of a 500 ms white-light flash, preceded and followed by a 500- and 1000-ms period of darkness, and was repeated 100 times. The checkerboard stimulus consisted of 72,000 spatial frames, presented at 30 frames/s, where each individual frame comprised a 24 × 24 array of non-overlapping 80 µm^2^ checkers when projected onto the retina. Each checker was either UV, green or black (in equal measure) and randomly assigned to each frame. In addition, each frame was randomly jittered along its x- and y-axis (−80 µm < x,y < 80 µm) to prevent the checker elements from forming ‘hard’ boundaries over time. This chromatic white-noise checkerboard stimulus was presented twice, giving a total presentation period of 80 min.

### 4.5. Data Analysis

Spontaneous spiking activity was recorded in darkness for at least 15 min. To identify RGCs which were firing in an oscillatory manner, the spontaneous activity of each cell was binned at 1 kHz, and then divided into 5 s non-overlapping epochs. Each epoch was baseline subtracted and the autocorrelation and power spectral density determined. These results were then used to calculate the average autocorrelation and power spectral density for each cell. Cells were classified as oscillating if (a) its power spectrum displayed a peak between 0.5–15 Hz that was more than 2 standard deviations greater than the mean power of frequencies between 0.5 and 30 Hz; (b) additional peaks were also present at, at least the first harmonic; (c) the autocorrelation had periodically varying sidelobes (Figure 1). For the full-field light-flash stimulus, the ON-OFF index was calculated as:

(2) $$I=\frac{\left(ON-OFF\right)}{\left(ON+OFF\right)}$$

where, for the 100 stimulus repeats, *ON* is the total number of spikes that occurred during the 100 ms period that commenced 50 ms after the onset of the light stimulus, and *OFF* is the total number of spikes that occurred during the 100 ms period that commenced 50 ms after the light offset. An *ON*-*OFF* index of 1 would indicate a perfect *ON* response and a perfect *OFF* response indicated by −1. To calculate a cell’s average response to the stimulus, its response to each trial was first binned at 100 Hz before averaging. Figure 2a shows the average response of every cell as heatmaps, ranked in descending order according to its *ON*-*OFF* index, for WT and *Nyx^nob^* mice.

The chromatic white-noise checkerboard stimulus was used to determine the receptive fields of individual RGCs. For each cell, the reversed spike-triggered average (rSTA) was calculated separately for the green and UV checkers, for the 10 stimulus frames that preceded each spike. Only the frame containing the largest rSTA peak, irrespective of the peak’s polarity or chromatic preference, was analyzed further. Cells where the largest rSTA peak, irrespective of the peak’s polarity, occurred for the green checker stimulus were classified as green-lighting preferring RGCs (G-RGCs). Similarly, cells were classified as UV-light preferring RGCs (UV-RGCs) if their largest rSTA peak, irrespective of the peak’s polarity, occurred for the UV checker stimulus. The receptive field size was defined as the 2D region where the rSTA was greater than 40% of its maximal value. Only cells whose peak rSTA value was more than 2 standard deviations greater than the corresponding frame’s average value were included in the analysis. Any rSTA showing receptive fields from more than one cell was also excluded from the analysis.

### 4.6. Statistical Analysis

SPSS Statistics 25 (IBM Corp., Armonk, NY, USA) was used for statistical analysis. As the distribution of receptive field sizes for several groups violated the assumption of normality (Shapiro-Wilk test), all statistical differences between groups were assessed using the Mann–Whitney U test. Furthermore, the median ± median absolute deviation is used as a measurement of the central tendency for receptive field size data sets.

## Data Availability

All data can be accessed via https://figshare.com/articles/dataset/Receptive_field_area_nyxnob_xlsx/19367888 (accessed on 14 March 2022).
